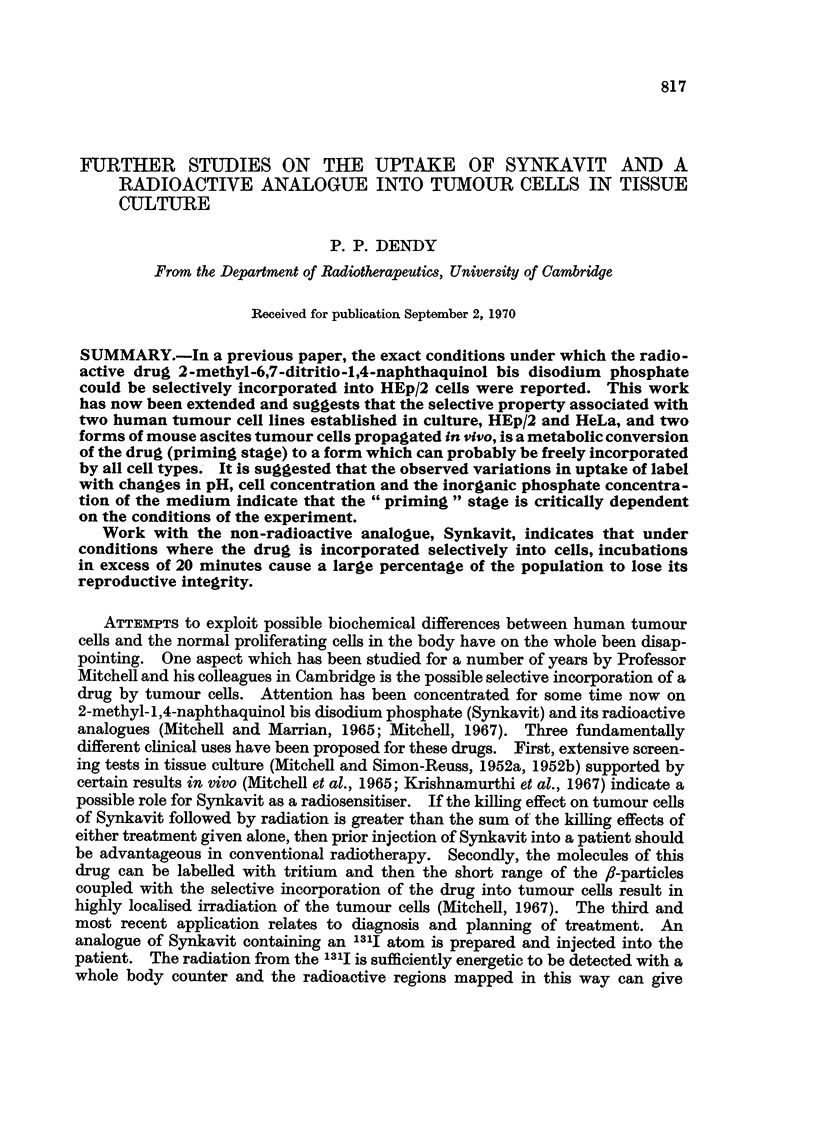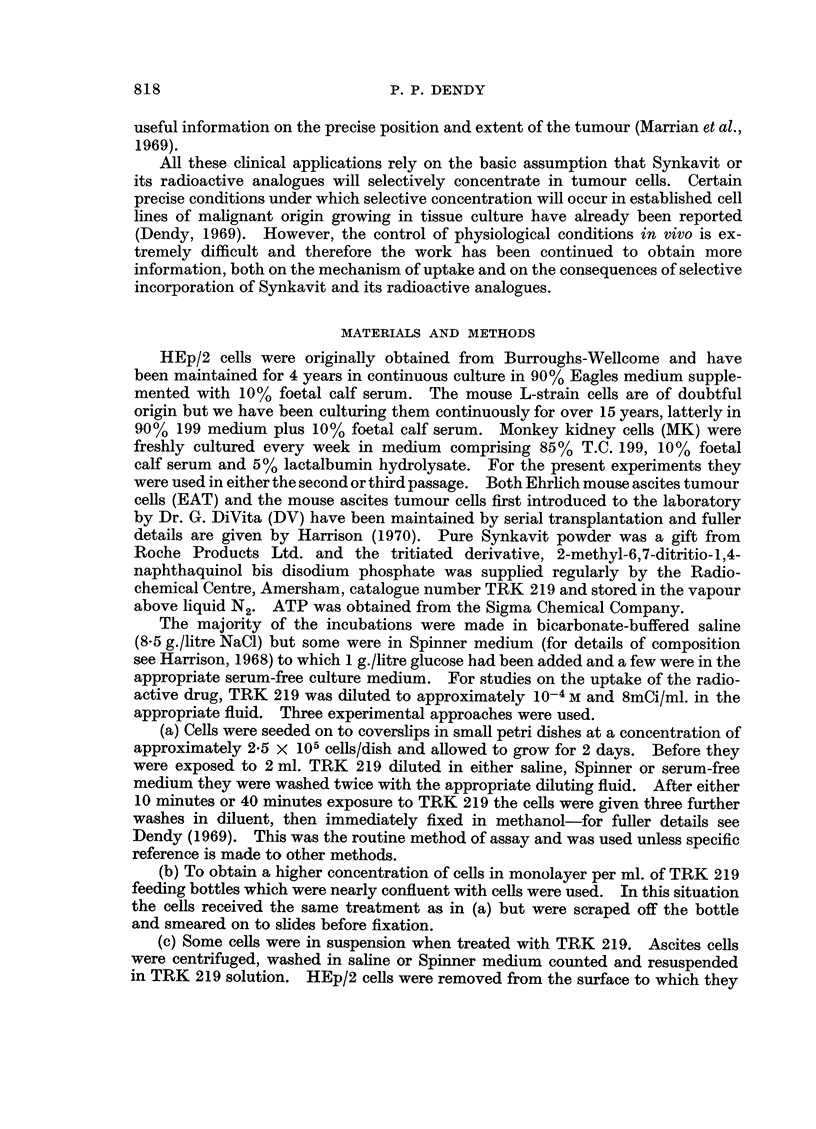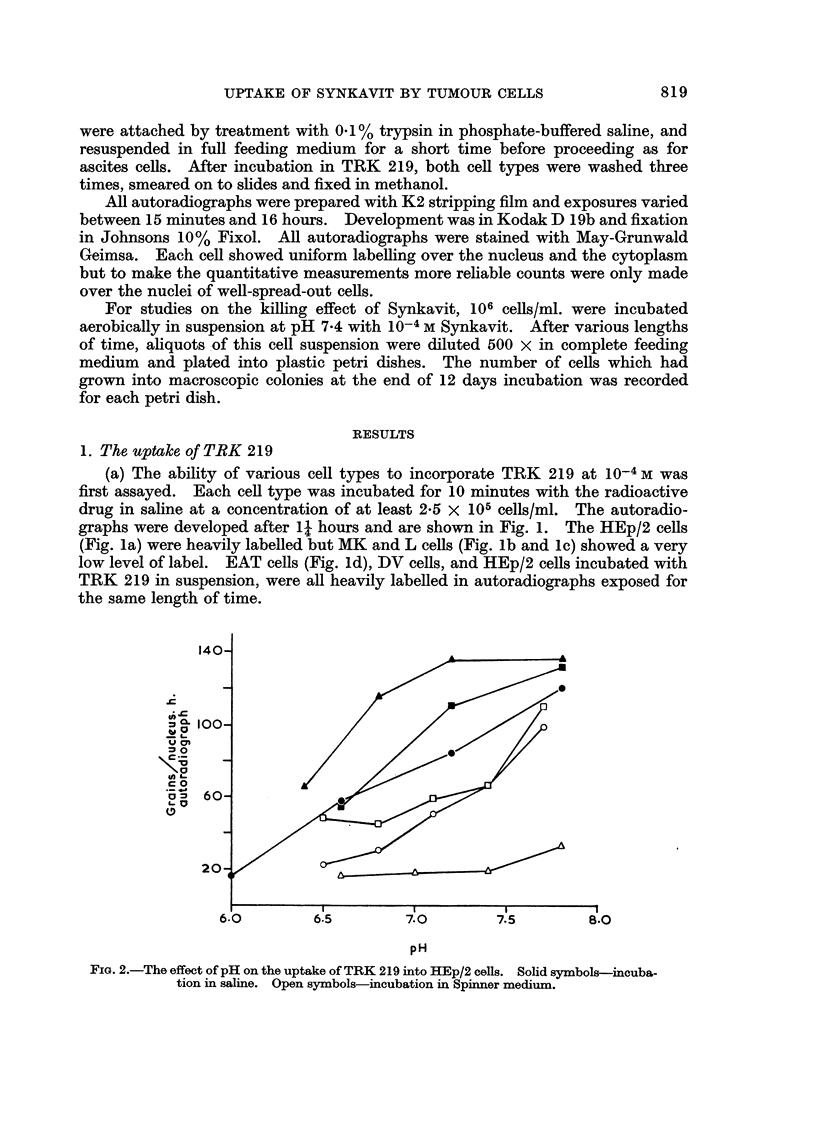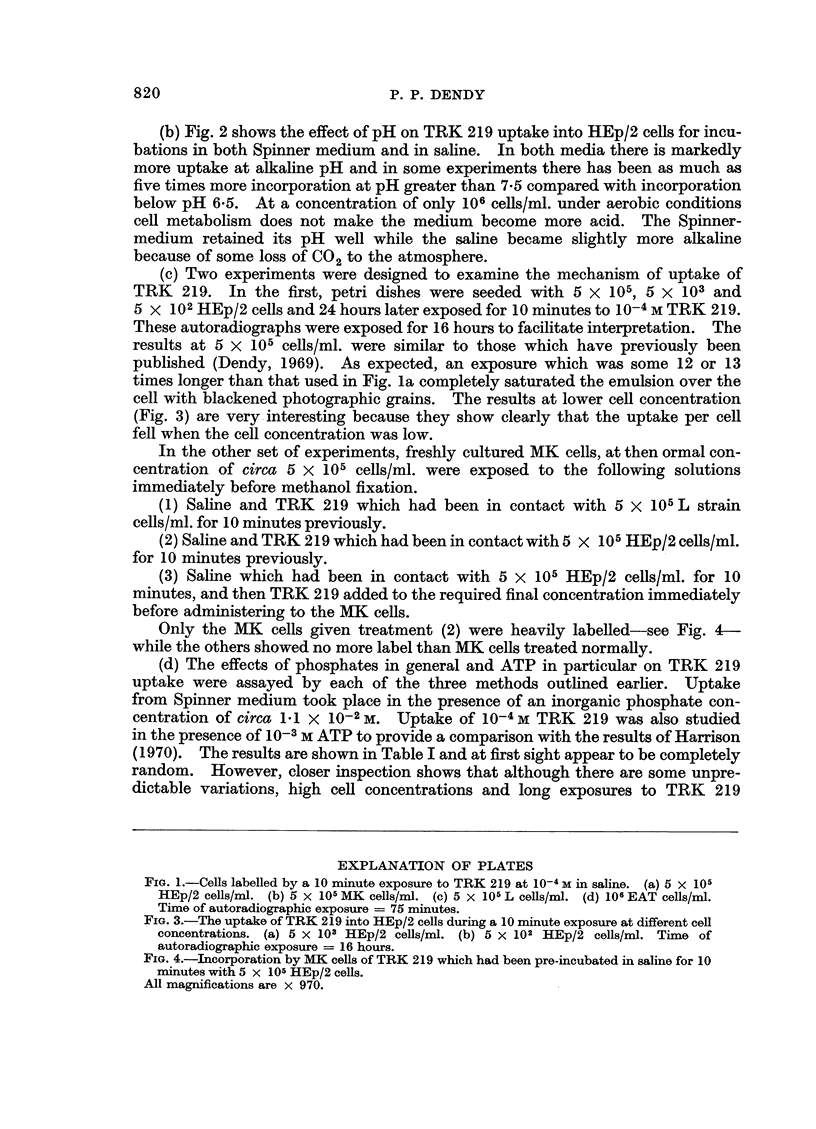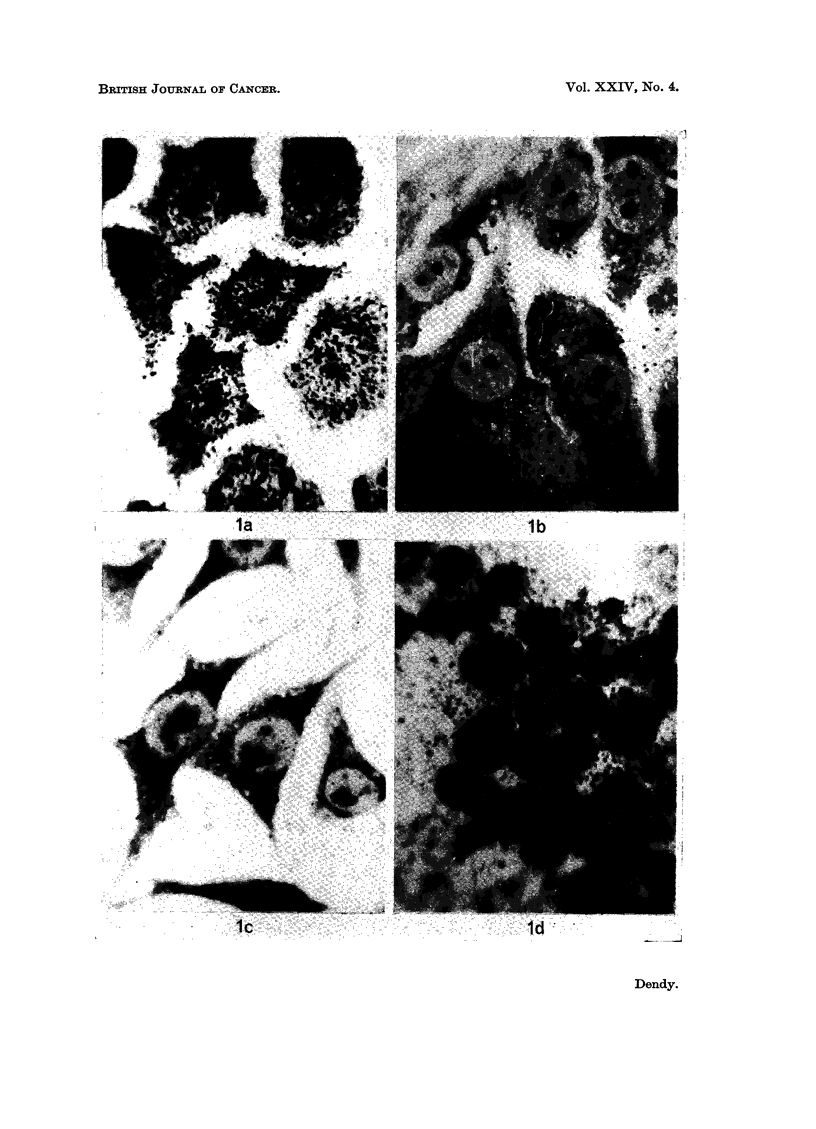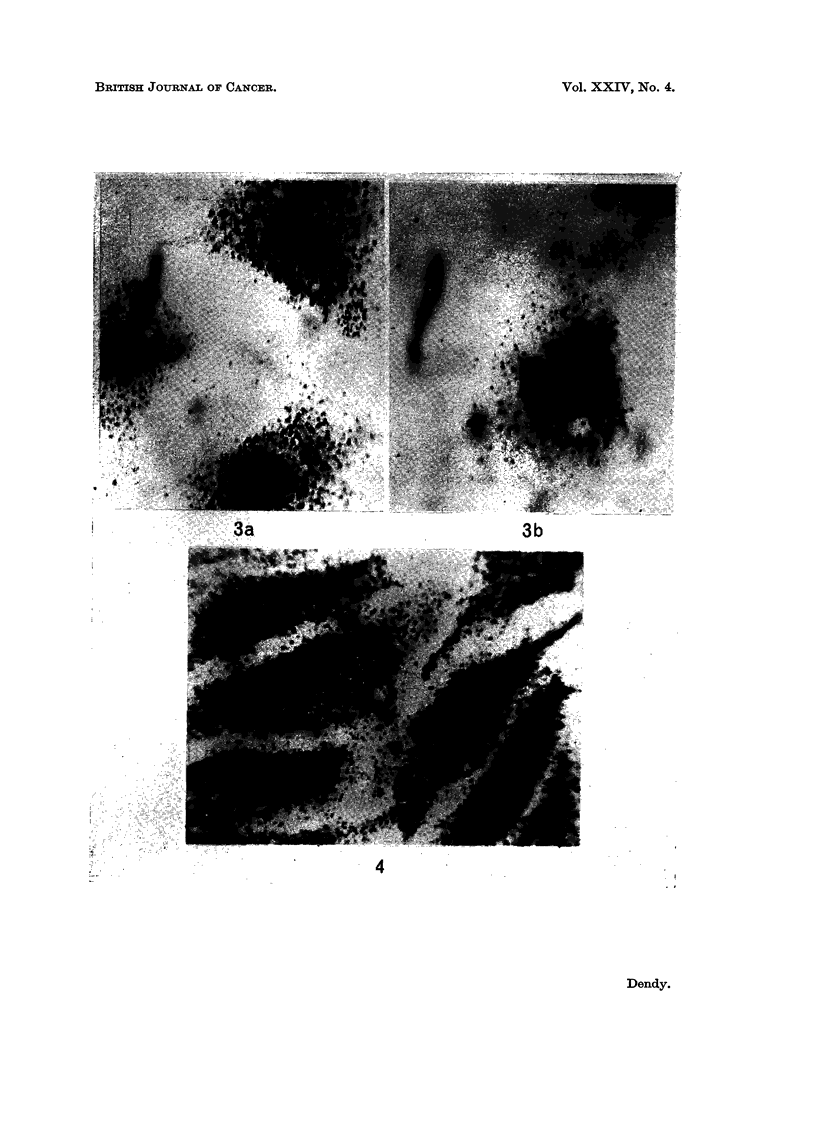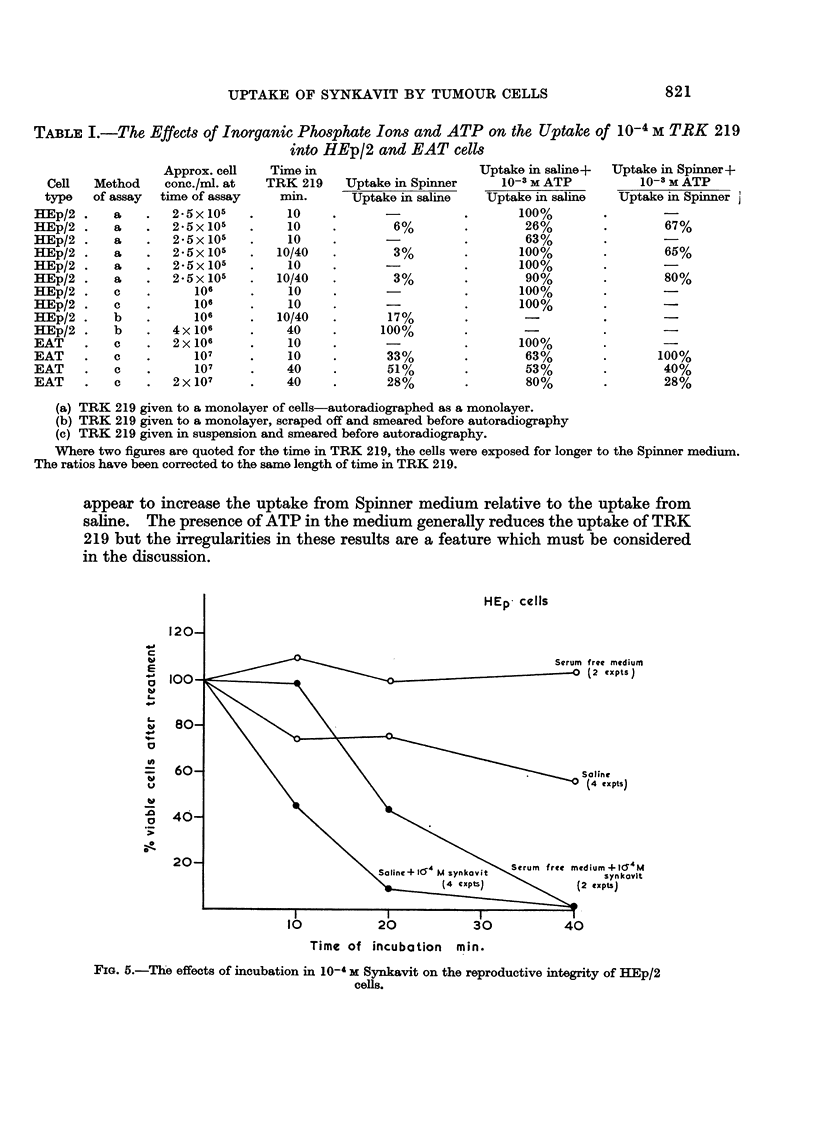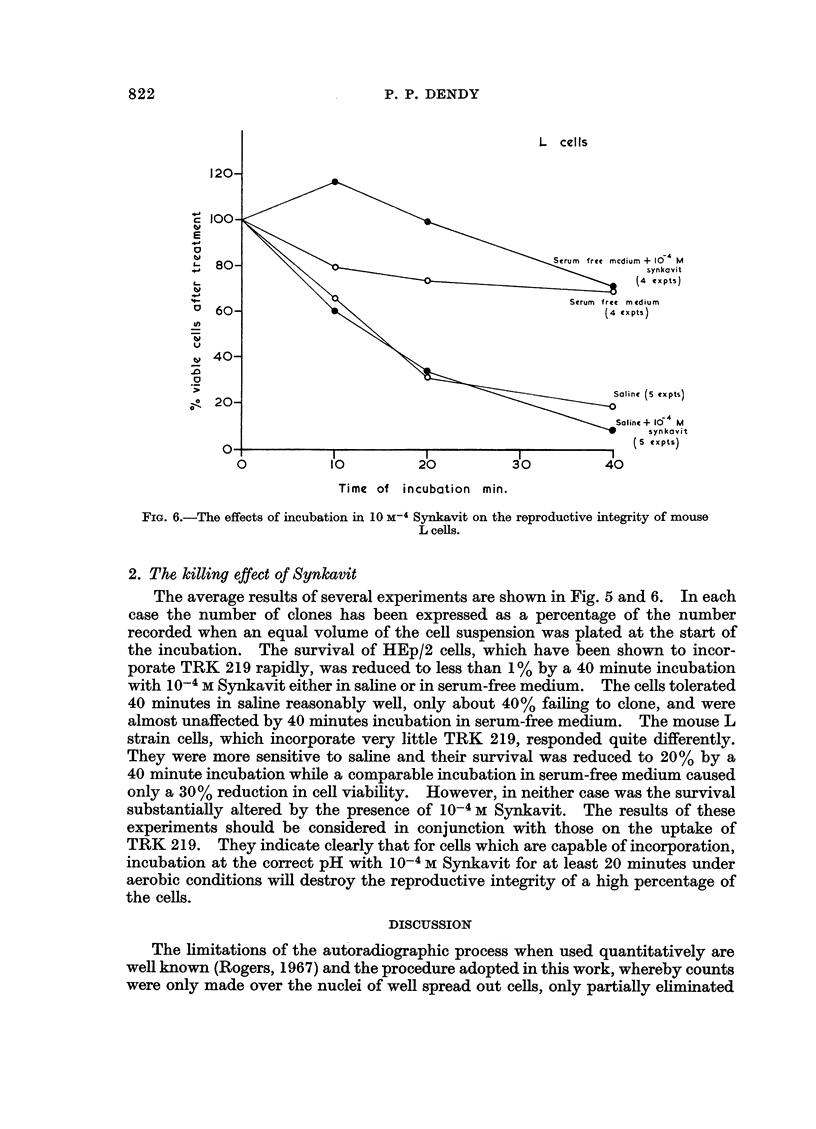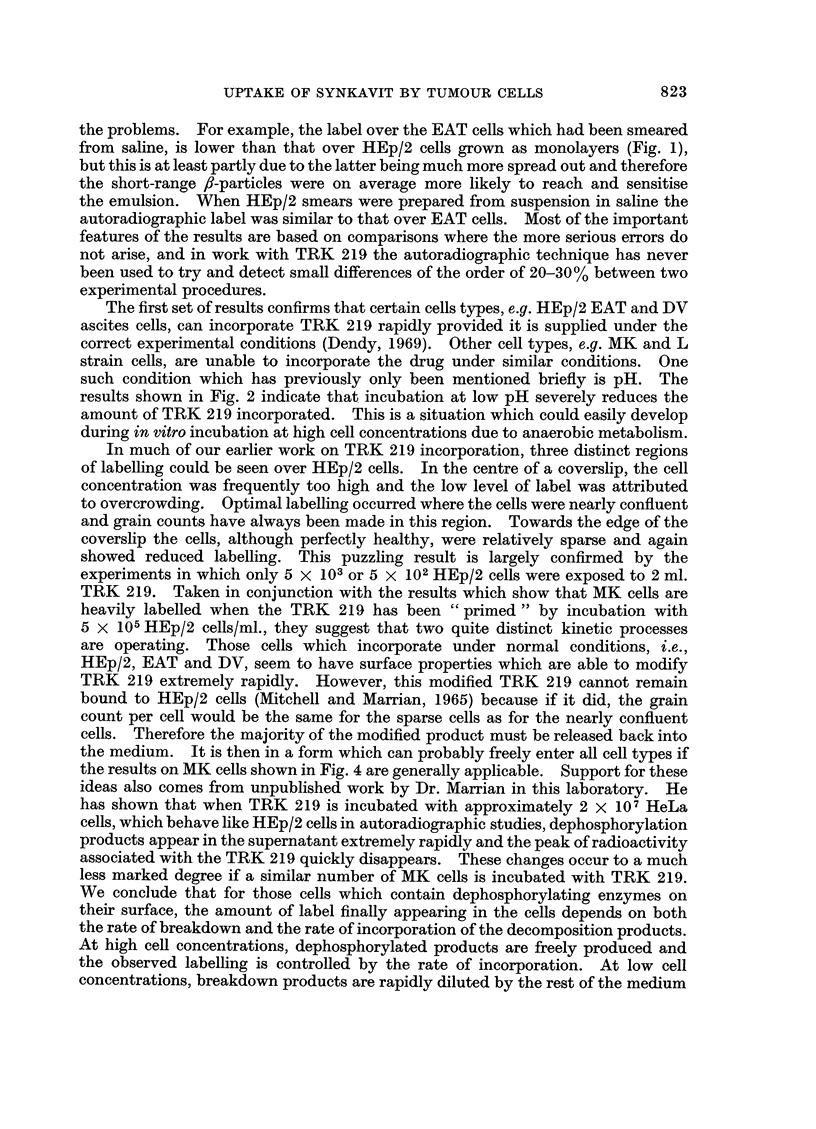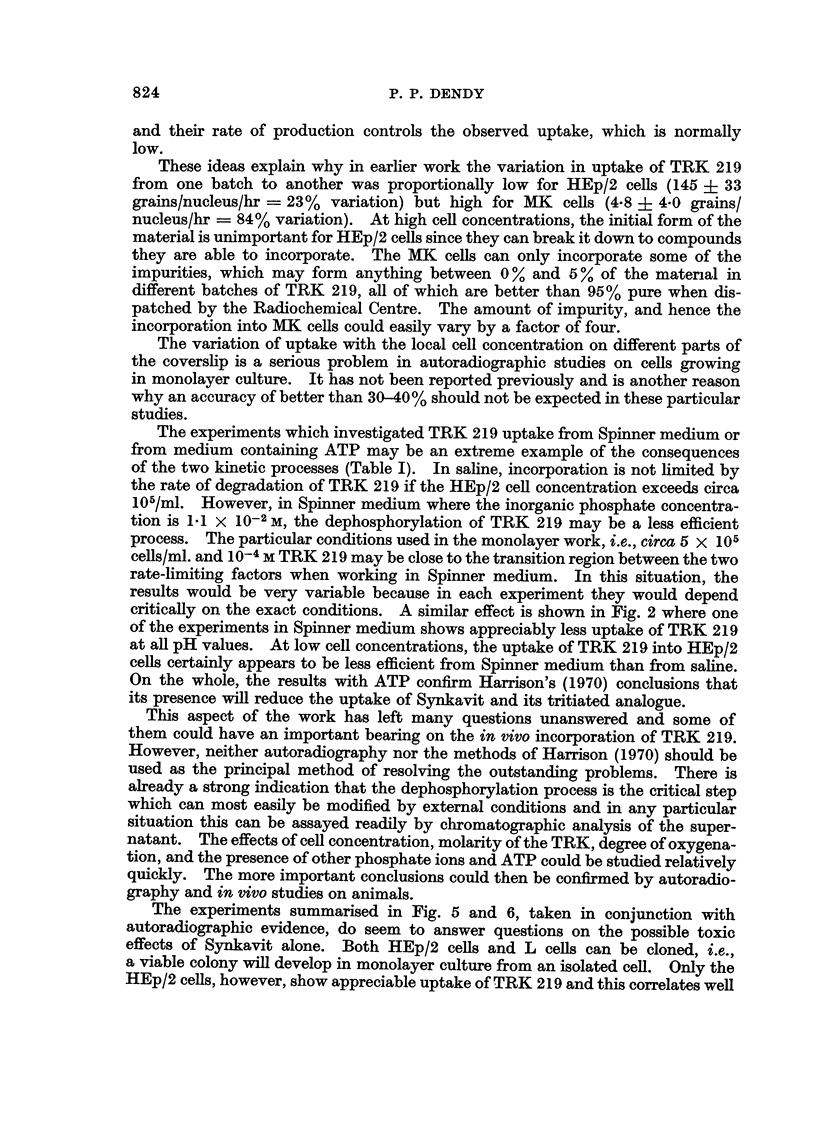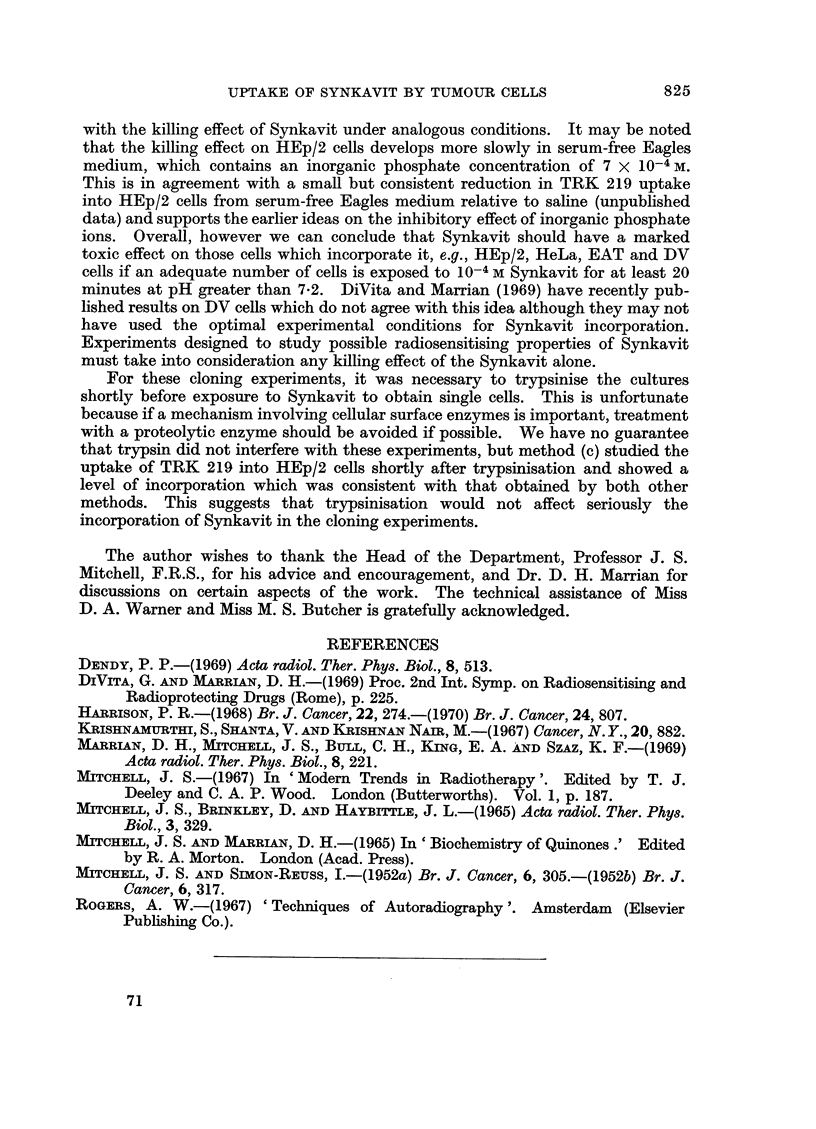# Further Studies on the Uptake of Synkavit and a Radioactive Analogue into Tumour Cells in Tissue Culture

**DOI:** 10.1038/bjc.1970.96

**Published:** 1970-12

**Authors:** P. P. Dendy

## Abstract

**Images:**


### FURTHER STUDIES ON THE UPTAKE OF SYNKAVIT AND A RADIOACTIVE ANALOGUE INTO TUMOUR CELLS IN TISSUE CULTURE

P. P. DENDY

*From the Department of Radiotherapeutics, University of Cambridge*

Received for publication September 2, 1970

**SUMMARY.—In a previous paper, the exact conditions under which the radioactive drug 2-methyl-6,7-ditritio-1,4-naphthaquinol bis disodium phosphate could be selectively incorporated into HEp/2 cells were reported. This work has now been extended and suggests that the selective property associated with two human tumour cell lines established in culture, HEp/2 and HeLa, and two forms of mouse ascites tumour cells propagated *in vivo*, is a metabolic conversion of the drug (priming stage) to a form which can probably be freely incorporated by all cell types. It is suggested that the observed variations in uptake of label with changes in pH, cell concentration and the inorganic phosphate concentration of the medium indicate that the " priming " stage is critically dependent on the conditions of the experiment.**

**Work with the non-radioactive analogue, Synkavit, indicates that under conditions where the drug is incorporated selectively into cells, incubations in excess of 20 minutes cause a large percentage of the population to lose its reproductive integrity.**

ATTEMPTS to exploit possible biochemical differences between human tumour cells and the normal proliferating cells in the body have on the whole been disappointing. One aspect which has been studied for a number of years by Professor Mitchell and his colleagues in Cambridge is the possible selective incorporation of a drug by tumour cells. Attention has been concentrated for some time now on 2-methyl-1,4-naphthaquinol bis disodium phosphate (Synkavit) and its radioactive analogues (Mitchell and Marrian, 1965; Mitchell, 1967). Three fundamentally different clinical uses have been proposed for these drugs. First, extensive screening tests in tissue culture (Mitchell and Simon-Reuss, 1952a, 1952b) supported by certain results *in vivo* (Mitchell *et al.*, 1965; Krishnamurthi *et al.*, 1967) indicate a possible role for Synkavit as a radiosensitiser. If the killing effect on tumour cells of Synkavit followed by radiation is greater than the sum of the killing effects of either treatment given alone, then prior injection of Synkavit into a patient should be advantageous in conventional radiotherapy. Secondly, the molecules of this drug can be labelled with tritium and then the short range of the $\beta$-particles coupled with the selective incorporation of the drug into tumour cells result in highly localised irradiation of the tumour cells (Mitchell, 1967). The third and most recent application relates to diagnosis and planning of treatment. An analogue of Synkavit containing an $^{131}$I atom is prepared and injected into the patient. The radiation from the $^{131}$I is sufficiently energetic to be detected with a whole body counter and the radioactive regions mapped in this way can give useful information on the precise position and extent of the tumour (Marrian *et al.*, 1969).

All these clinical applications rely on the basic assumption that Synkavit or its radioactive analogues will selectively concentrate in tumour cells. Certain precise conditions under which selective concentration will occur in established cell lines of malignant origin growing in tissue culture have already been reported (Dendy, 1969). However, the control of physiological conditions *in vivo* is extremely difficult and therefore the work has been continued to obtain more information, both on the mechanism of uptake and on the consequences of selective incorporation of Synkavit and its radioactive analogues.

### MATERIALS AND METHODS

HEp/2 cells were originally obtained from Burroughs-Wellcome and have been maintained for 4 years in continuous culture in 90% Eagles medium supplemented with 10% foetal calf serum. The mouse L-strain cells are of doubtful origin but we have been culturing them continuously for over 15 years, latterly in 90% 199 medium plus 10% foetal calf serum. Monkey kidney cells (MK) were freshly cultured every week in medium comprising 85% T.C. 199, 10% foetal calf serum and 5% lactalbumin hydrolysate. For the present experiments they were used in either the second or third passage. Both Ehrlich mouse ascites tumour cells (EAT) and the mouse ascites tumour cells first introduced to the laboratory by Dr. G. DiVita (DV) have been maintained by serial transplantation and fuller details are given by Harrison (1970). Pure Synkavit powder was a gift from Roche Products Ltd. and the tritiated derivative, 2-methyl-6,7-ditritio-1,4-naphthaquinol bis disodium phosphate was supplied regularly by the Radiochemical Centre, Amersham, catalogue number TRK 219 and stored in the vapour above liquid $N_2$. ATP was obtained from the Sigma Chemical Company.

The majority of the incubations were made in bicarbonate-buffered saline (8·5 g./litre NaCl) but some were in Spinner medium (for details of composition see Harrison, 1968) to which 1 g./litre glucose had been added and a few were in the appropriate serum-free culture medium. For studies on the uptake of the radioactive drug, TRK 219 was diluted to approximately $10^{-4}$ M and 8mCi/ml. in the appropriate fluid. Three experimental approaches were used.

(a) Cells were seeded on to coverslips in small petri dishes at a concentration of approximately $2{\cdot}5 \times 10^5$ cells/dish and allowed to grow for 2 days. Before they were exposed to 2 ml. TRK 219 diluted in either saline, Spinner or serum-free medium they were washed twice with the appropriate diluting fluid. After either 10 minutes or 40 minutes exposure to TRK 219 the cells were given three further washes in diluent, then immediately fixed in methanol—for fuller details see Dendy (1969). This was the routine method of assay and was used unless specific reference is made to other methods.

(b) To obtain a higher concentration of cells in monolayer per ml. of TRK 219 feeding bottles which were nearly confluent with cells were used. In this situation the cells received the same treatment as in (a) but were scraped off the bottle and smeared on to slides before fixation.

(c) Some cells were in suspension when treated with TRK 219. Ascites cells were centrifuged, washed in saline or Spinner medium counted and resuspended in TRK 219 solution. HEp/2 cells were removed from the surface to which they were attached by treatment with 0·1% trypsin in phosphate-buffered saline, and resuspended in full feeding medium for a short time before proceeding as for ascites cells. After incubation in TRK 219, both cell types were washed three times, smeared on to slides and fixed in methanol.

All autoradiographs were prepared with K2 stripping film and exposures varied between 15 minutes and 16 hours. Development was in Kodak D 19b and fixation in Johnsons 10% Fixol. All autoradiographs were stained with May-Grunwald Geimsa. Each cell showed uniform labelling over the nucleus and the cytoplasm but to make the quantitative measurements more reliable counts were only made over the nuclei of well-spread-out cells.

For studies on the killing effect of Synkavit, $10^6$ cells/ml. were incubated aerobically in suspension at pH 7·4 with $10^{-4}$ M Synkavit. After various lengths of time, aliquots of this cell suspension were diluted 500 × in complete feeding medium and plated into plastic petri dishes. The number of cells which had grown into macroscopic colonies at the end of 12 days incubation was recorded for each petri dish.

### RESULTS

### 1. *The uptake of TRK* 219

(a) The ability of various cell types to incorporate TRK 219 at $10^{-4}$ M was first assayed. Each cell type was incubated for 10 minutes with the radioactive drug in saline at a concentration of at least 2·5 × $10^5$ cells/ml. The autoradiographs were developed after 1¼ hours and are shown in Fig. 1. The HEp/2 cells (Fig. 1a) were heavily labelled but MK and L cells (Fig. 1b and 1c) showed a very low level of label. EAT cells (Fig. 1d), DV cells, and HEp/2 cells incubated with TRK 219 in suspension, were all heavily labelled in autoradiographs exposed for the same length of time.

FIG. 2.—The effect of pH on the uptake of TRK 219 into HEp/2 cells. Solid symbols—incubation in saline. Open symbols—incubation in Spinner medium.

(b) Fig. 2 shows the effect of pH on TRK 219 uptake into HEp/2 cells for incubations in both Spinner medium and in saline. In both media there is markedly more uptake at alkaline pH and in some experiments there has been as much as five times more incorporation at pH greater than 7·5 compared with incorporation below pH 6·5. At a concentration of only $10^6$ cells/ml. under aerobic conditions cell metabolism does not make the medium become more acid. The Spinner-medium retained its pH well while the saline became slightly more alkaline because of some loss of $CO_2$ to the atmosphere.

(c) Two experiments were designed to examine the mechanism of uptake of TRK 219. In the first, petri dishes were seeded with $5 \times 10^5$, $5 \times 10^3$ and $5 \times 10^2$ HEp/2 cells and 24 hours later exposed for 10 minutes to $10^{-4}$ M TRK 219. These autoradiographs were exposed for 16 hours to facilitate interpretation. The results at $5 \times 10^5$ cells/ml. were similar to those which have previously been published (Dendy, 1969). As expected, an exposure which was some 12 or 13 times longer than that used in Fig. 1a completely saturated the emulsion over the cell with blackened photographic grains. The results at lower cell concentration (Fig. 3) are very interesting because they show clearly that the uptake per cell fell when the cell concentration was low.

In the other set of experiments, freshly cultured MK cells, at then ormal concentration of *circa* $5 \times 10^5$ cells/ml. were exposed to the following solutions immediately before methanol fixation.

(1) Saline and TRK 219 which had been in contact with $5 \times 10^5$ L strain cells/ml. for 10 minutes previously.

(2) Saline and TRK 219 which had been in contact with $5 \times 10^5$ HEp/2 cells/ml. for 10 minutes previously.

(3) Saline which had been in contact with $5 \times 10^5$ HEp/2 cells/ml. for 10 minutes, and then TRK 219 added to the required final concentration immediately before administering to the MK cells.

Only the MK cells given treatment (2) were heavily labelled—see Fig. 4—while the others showed no more label than MK cells treated normally.

(d) The effects of phosphates in general and ATP in particular on TRK 219 uptake were assayed by each of the three methods outlined earlier. Uptake from Spinner medium took place in the presence of an inorganic phosphate concentration of *circa* $1{\cdot}1 \times 10^{-2}$ M. Uptake of $10^{-4}$ M TRK 219 was also studied in the presence of $10^{-3}$ M ATP to provide a comparison with the results of Harrison (1970). The results are shown in Table I and at first sight appear to be completely random. However, closer inspection shows that although there are some unpredictable variations, high cell concentrations and long exposures to TRK 219

---

EXPLANATION OF PLATES

Fig. 1.—Cells labelled by a 10 minute exposure to TRK 219 at $10^{-4}$ M in saline. (a) $5 \times 10^5$ HEp/2 cells/ml. (b) $5 \times 10^5$ MK cells/ml. (c) $5 \times 10^5$ L cells/ml. (d) $10^6$ EAT cells/ml. Time of autoradiographic exposure = 75 minutes.

Fig. 3.—The uptake of TRK 219 into HEp/2 cells during a 10 minute exposure at different cell concentrations. (a) $5 \times 10^3$ HEp/2 cells/ml. (b) $5 \times 10^2$ HEp/2 cells/ml. Time of autoradiographic exposure = 16 hours.

Fig. 4.—Incorporation by MK cells of TRK 219 which had been pre-incubated in saline for 10 minutes with $5 \times 10^5$ HEp/2 cells.

All magnifications are × 970.

Dendy.

3a 3b

4

Dendy.

TABLE I.—*The Effects of Inorganic Phosphate Ions and ATP on the Uptake of* $10^{-4}$ M *TRK* 219 *into HEp/2 and EAT cells*

| Cell type | Method of assay | Approx. cell conc./ml. at time of assay | Time in TRK 219 min. | Uptake in Spinner / Uptake in saline | Uptake in saline + $10^{-3}$ M ATP / Uptake in saline | Uptake in Spinner + $10^{-3}$ M ATP / Uptake in Spinner |
|---|---|---|---|---|---|---|
| HEp/2 | a | $2\cdot5\times10^5$ | 10 | — | 100% | — |
| HEp/2 | a | $2\cdot5\times10^5$ | 10 | 6% | 26% | 67% |
| HEp/2 | a | $2\cdot5\times10^5$ | 10 | — | 63% | — |
| HEp/2 | a | $2\cdot5\times10^5$ | 10/40 | 3% | 100% | 65% |
| HEp/2 | a | $2\cdot5\times10^5$ | 10 | — | 100% | — |
| HEp/2 | a | $2\cdot5\times10^5$ | 10/40 | 3% | 90% | 80% |
| HEp/2 | c | $10^6$ | 10 | — | 100% | — |
| HEp/2 | c | $10^6$ | 10 | — | 100% | — |
| HEp/2 | b | $10^6$ | 10/40 | 17% | — | — |
| HEp/2 | b | $4\times10^6$ | 40 | 100% | — | — |
| EAT | c | $2\times10^6$ | 10 | — | 100% | — |
| EAT | c | $10^7$ | 10 | 33% | 63% | 100% |
| EAT | c | $10^7$ | 40 | 51% | 53% | 40% |
| EAT | c | $2\times10^7$ | 40 | 28% | 80% | 28% |

(a) TRK 219 given to a monolayer of cells—autoradiographed as a monolayer.
(b) TRK 219 given to a monolayer, scraped off and smeared before autoradiography
(c) TRK 219 given in suspension and smeared before autoradiography.

Where two figures are quoted for the time in TRK 219, the cells were exposed for longer to the Spinner medium. The ratios have been corrected to the same length of time in TRK 219.

appear to increase the uptake from Spinner medium relative to the uptake from saline. The presence of ATP in the medium generally reduces the uptake of TRK 219 but the irregularities in these results are a feature which must be considered in the discussion.

FIG. 5.—The effects of incubation in $10^{-4}$ M Synkavit on the reproductive integrity of HEp/2 cells.

FIG. 6.—The effects of incubation in 10 $M^{-4}$ Synkavit on the reproductive integrity of mouse L cells.

### 2. *The killing effect of Synkavit*

The average results of several experiments are shown in Fig. 5 and 6. In each case the number of clones has been expressed as a percentage of the number recorded when an equal volume of the cell suspension was plated at the start of the incubation. The survival of HEp/2 cells, which have been shown to incorporate TRK 219 rapidly, was reduced to less than 1% by a 40 minute incubation with $10^{-4}$ M Synkavit either in saline or in serum-free medium. The cells tolerated 40 minutes in saline reasonably well, only about 40% failing to clone, and were almost unaffected by 40 minutes incubation in serum-free medium. The mouse L strain cells, which incorporate very little TRK 219, responded quite differently. They were more sensitive to saline and their survival was reduced to 20% by a 40 minute incubation while a comparable incubation in serum-free medium caused only a 30% reduction in cell viability. However, in neither case was the survival substantially altered by the presence of $10^{-4}$ M Synkavit. The results of these experiments should be considered in conjunction with those on the uptake of TRK 219. They indicate clearly that for cells which are capable of incorporation, incubation at the correct pH with $10^{-4}$ M Synkavit for at least 20 minutes under aerobic conditions will destroy the reproductive integrity of a high percentage of the cells.

### DISCUSSION

The limitations of the autoradiographic process when used quantitatively are well known (Rogers, 1967) and the procedure adopted in this work, whereby counts were only made over the nuclei of well spread out cells, only partially eliminated the problems. For example, the label over the EAT cells which had been smeared from saline, is lower than that over HEp/2 cells grown as monolayers (Fig. 1), but this is at least partly due to the latter being much more spread out and therefore the short-range $\beta$-particles were on average more likely to reach and sensitise the emulsion. When HEp/2 smears were prepared from suspension in saline the autoradiographic label was similar to that over EAT cells. Most of the important features of the results are based on comparisons where the more serious errors do not arise, and in work with TRK 219 the autoradiographic technique has never been used to try and detect small differences of the order of 20–30% between two experimental procedures.

The first set of results confirms that certain cells types, *e.g.* HEp/2 EAT and DV ascites cells, can incorporate TRK 219 rapidly provided it is supplied under the correct experimental conditions (Dendy, 1969). Other cell types, *e.g.* MK and L strain cells, are unable to incorporate the drug under similar conditions. One such condition which has previously only been mentioned briefly is pH. The results shown in Fig. 2 indicate that incubation at low pH severely reduces the amount of TRK 219 incorporated. This is a situation which could easily develop during *in vitro* incubation at high cell concentrations due to anaerobic metabolism.

In much of our earlier work on TRK 219 incorporation, three distinct regions of labelling could be seen over HEp/2 cells. In the centre of a coverslip, the cell concentration was frequently too high and the low level of label was attributed to overcrowding. Optimal labelling occurred where the cells were nearly confluent and grain counts have always been made in this region. Towards the edge of the coverslip the cells, although perfectly healthy, were relatively sparse and again showed reduced labelling. This puzzling result is largely confirmed by the experiments in which only $5 \times 10^3$ or $5 \times 10^2$ HEp/2 cells were exposed to 2 ml. TRK 219. Taken in conjunction with the results which show that MK cells are heavily labelled when the TRK 219 has been "primed" by incubation with $5 \times 10^5$ HEp/2 cells/ml., they suggest that two quite distinct kinetic processes are operating. Those cells which incorporate under normal conditions, *i.e.*, HEp/2, EAT and DV, seem to have surface properties which are able to modify TRK 219 extremely rapidly. However, this modified TRK 219 cannot remain bound to HEp/2 cells (Mitchell and Marrian, 1965) because if it did, the grain count per cell would be the same for the sparse cells as for the nearly confluent cells. Therefore the majority of the modified product must be released back into the medium. It is then in a form which can probably freely enter all cell types if the results on MK cells shown in Fig. 4 are generally applicable. Support for these ideas also comes from unpublished work by Dr. Marrian in this laboratory. He has shown that when TRK 219 is incubated with approximately $2 \times 10^7$ HeLa cells, which behave like HEp/2 cells in autoradiographic studies, dephosphorylation products appear in the supernatant extremely rapidly and the peak of radioactivity associated with the TRK 219 quickly disappears. These changes occur to a much less marked degree if a similar number of MK cells is incubated with TRK 219. We conclude that for those cells which contain dephosphorylating enzymes on their surface, the amount of label finally appearing in the cells depends on both the rate of breakdown and the rate of incorporation of the decomposition products. At high cell concentrations, dephosphorylated products are freely produced and the observed labelling is controlled by the rate of incorporation. At low cell concentrations, breakdown products are rapidly diluted by the rest of the medium and their rate of production controls the observed uptake, which is normally low.

These ideas explain why in earlier work the variation in uptake of TRK 219 from one batch to another was proportionally low for HEp/2 cells ($145 \pm 33$ grains/nucleus/hr = 23% variation) but high for MK cells ($4{\cdot}8 \pm 4{\cdot}0$ grains/nucleus/hr = 84% variation). At high cell concentrations, the initial form of the material is unimportant for HEp/2 cells since they can break it down to compounds they are able to incorporate. The MK cells can only incorporate some of the impurities, which may form anything between 0% and 5% of the material in different batches of TRK 219, all of which are better than 95% pure when dispatched by the Radiochemical Centre. The amount of impurity, and hence the incorporation into MK cells could easily vary by a factor of four.

The variation of uptake with the local cell concentration on different parts of the coverslip is a serious problem in autoradiographic studies on cells growing in monolayer culture. It has not been reported previously and is another reason why an accuracy of better than 30–40% should not be expected in these particular studies.

The experiments which investigated TRK 219 uptake from Spinner medium or from medium containing ATP may be an extreme example of the consequences of the two kinetic processes (Table I). In saline, incorporation is not limited by the rate of degradation of TRK 219 if the HEp/2 cell concentration exceeds circa $10^5$/ml. However, in Spinner medium where the inorganic phosphate concentration is $1{\cdot}1 \times 10^{-2}$ M, the dephosphorylation of TRK 219 may be a less efficient process. The particular conditions used in the monolayer work, *i.e.*, *circa* $5 \times 10^5$ cells/ml. and $10^{-4}$ M TRK 219 may be close to the transition region between the two rate-limiting factors when working in Spinner medium. In this situation, the results would be very variable because in each experiment they would depend critically on the exact conditions. A similar effect is shown in Fig. 2 where one of the experiments in Spinner medium shows appreciably less uptake of TRK 219 at all pH values. At low cell concentrations, the uptake of TRK 219 into HEp/2 cells certainly appears to be less efficient from Spinner medium than from saline. On the whole, the results with ATP confirm Harrison's (1970) conclusions that its presence will reduce the uptake of Synkavit and its tritiated analogue.

This aspect of the work has left many questions unanswered and some of them could have an important bearing on the *in vivo* incorporation of TRK 219. However, neither autoradiography nor the methods of Harrison (1970) should be used as the principal method of resolving the outstanding problems. There is already a strong indication that the dephosphorylation process is the critical step which can most easily be modified by external conditions and in any particular situation this can be assayed readily by chromatographic analysis of the supernatant. The effects of cell concentration, molarity of the TRK, degree of oxygenation, and the presence of other phosphate ions and ATP could be studied relatively quickly. The more important conclusions could then be confirmed by autoradiography and *in vivo* studies on animals.

The experiments summarised in Fig. 5 and 6, taken in conjunction with autoradiographic evidence, do seem to answer questions on the possible toxic effects of Synkavit alone. Both HEp/2 cells and L cells can be cloned, *i.e.*, a viable colony will develop in monolayer culture from an isolated cell. Only the HEp/2 cells, however, show appreciable uptake of TRK 219 and this correlates well with the killing effect of Synkavit under analogous conditions. It may be noted that the killing effect on HEp/2 cells develops more slowly in serum-free Eagles medium, which contains an inorganic phosphate concentration of $7 \times 10^{-4}$ M. This is in agreement with a small but consistent reduction in TRK 219 uptake into HEp/2 cells from serum-free Eagles medium relative to saline (unpublished data) and supports the earlier ideas on the inhibitory effect of inorganic phosphate ions. Overall, however we can conclude that Synkavit should have a marked toxic effect on those cells which incorporate it, *e.g.*, HEp/2, HeLa, EAT and DV cells if an adequate number of cells is exposed to $10^{-4}$ M Synkavit for at least 20 minutes at pH greater than 7·2. DiVita and Marrian (1969) have recently published results on DV cells which do not agree with this idea although they may not have used the optimal experimental conditions for Synkavit incorporation. Experiments designed to study possible radiosensitising properties of Synkavit must take into consideration any killing effect of the Synkavit alone.

For these cloning experiments, it was necessary to trypsinise the cultures shortly before exposure to Synkavit to obtain single cells. This is unfortunate because if a mechanism involving cellular surface enzymes is important, treatment with a proteolytic enzyme should be avoided if possible. We have no guarantee that trypsin did not interfere with these experiments, but method (c) studied the uptake of TRK 219 into HEp/2 cells shortly after trypsinisation and showed a level of incorporation which was consistent with that obtained by both other methods. This suggests that trypsinisation would not affect seriously the incorporation of Synkavit in the cloning experiments.

The author wishes to thank the Head of the Department, Professor J. S. Mitchell, F.R.S., for his advice and encouragement, and Dr. D. H. Marrian for discussions on certain aspects of the work. The technical assistance of Miss D. A. Warner and Miss M. S. Butcher is gratefully acknowledged.

### REFERENCES

DENDY, P. P.—(1969) *Acta radiol. Ther. Phys. Biol.*, **8**, 513.

DIVITA, G. AND MARRIAN, D. H.—(1969) Proc. 2nd Int. Symp. on Radiosensitising and Radioprotecting Drugs (Rome), p. 225.

HARRISON, P. R.—(1968) *Br. J. Cancer*, **22**, 274.—(1970) *Br. J. Cancer*, **24**, 807.

KRISHNAMURTHI, S., SHANTA, V. AND KRISHNAN NAIR, M.—(1967) *Cancer, N.Y.*, **20**, 882.

MARRIAN, D. H., MITCHELL, J. S., BULL, C. H., KING, E. A. AND SZAZ, K. F.—(1969) *Acta radiol. Ther. Phys. Biol.*, **8**, 221.

MITCHELL, J. S.—(1967) In ‘Modern Trends in Radiotherapy’. Edited by T. J. Deeley and C. A. P. Wood. London (Butterworths). Vol. 1, p. 187.

MITCHELL, J. S., BRINKLEY, D. AND HAYBITTLE, J. L.—(1965) *Acta radiol. Ther. Phys. Biol.*, **3**, 329.

MITCHELL, J. S. AND MARRIAN, D. H.—(1965) In ‘Biochemistry of Quinones.’ Edited by R. A. Morton. London (Acad. Press).

MITCHELL, J. S. AND SIMON-REUSS, I.—(1952*a*) *Br. J. Cancer*, **6**, 305.—(1952*b*) *Br. J. Cancer*, **6**, 317.

ROGERS, A. W.—(1967) ‘Techniques of Autoradiography’. Amsterdam (Elsevier Publishing Co.).